# Protocol for detecting and quantifying hATF4-HA in non-stress versus stress conditions using automated and quantitative Jess western blotting

**DOI:** 10.1016/j.xpro.2024.103454

**Published:** 2024-11-20

**Authors:** Anna M. Smirnova, Anna Herrmannová, Leoš Shivaya Valášek

**Affiliations:** 1 Laboratory of Regulation of Gene Expression, Institute of Microbiology of the Czech Academy of Sciences, Videnska 1083, Prague 142 20, Czech Republic

**Keywords:** cell culture, gene expression, protein expression and purification

## Abstract

Activating transcription factor 4 (ATF4) is a key player in the integrated stress response, whose expression is subject to tight translational control. Studying its stress-provoked induction, accompanied by the general translational shutdown, is intricate because the expression of reference genes declines rapidly, and finding appropriate normalization controls is challenging. We present a protocol for human hemagglutinin-tagged ATF4 (hATF4-HA) detection and high-throughput quantification in non-stress versus stress conditions using automated and quantitative western blotting. We describe steps for seeding cells, transfecting plasmids, thapsigargin treatment, sample preparation, and target protein detection.

For complete details on the use and execution of this protocol, please refer to Smirnova et al.^1^

## Highlights

• Step-by-step guide for the detection and quantification of human ATF4-HA tag in Jess
• Protocol for sample preparation from transfected, thapsigargin-treated HEK293T cells
• Guidance on control steps for accurate estimation of ATF4-HA tag levels in samples
• Detailed explanation of data analysis and interpretation of Jess results

## Abstract (editor highlights)

Publisher’s note: Undertaking any experimental protocol requires adherence to local institutional guidelines for laboratory safety and ethics.

## Graphical abstract

**Figure:**
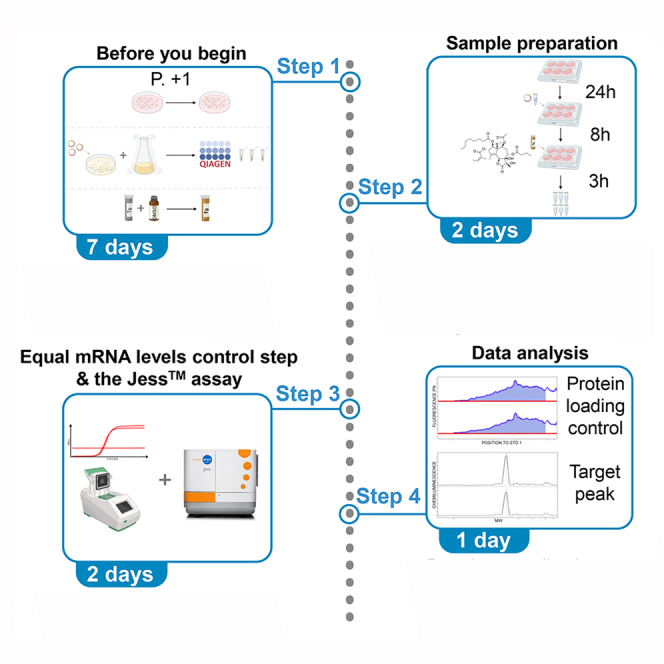

## Before you begin

### Cells preparation

**Timing: 7 days**

1. Order a cryovial of frozen HEK293T cells from your supplier (e.g., ATCC).

***Note:*** In this paragraph, we present a procedure for culturing human cell line HEK293T. We start with a cryovial of frozen HEK293T cells and finish with about 6 × 10^6^ cells.

2. Prepare a workspace for thawing a frozen HEK293T cell line.
  a. Sterilize the biological safety cabinet class II (Thermo Scientific) using an Ultraviolet (UV) light.
  b. Prewarm high glucose Dulbecco’s modified Eagle’s Medium (DMEM) supplemented with 10% fetal bovine serum (FBS) at 37.0°C in a water bath.
  c. Pre-fill a tissue culture dish 100 (60.1 cm^2^) with 10 mL of pre-warmed medium.

**Figure 1:**
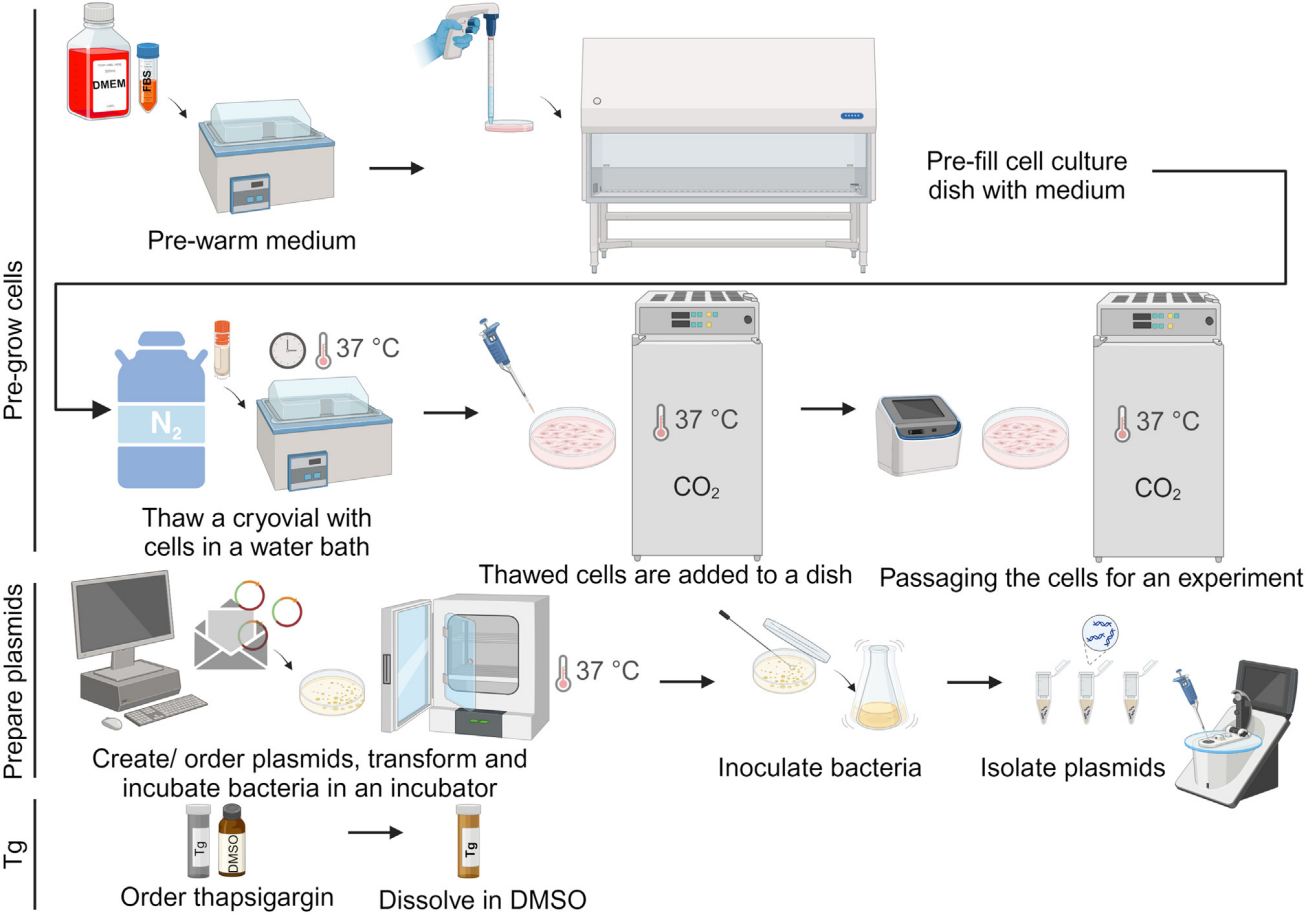
Before you begin: an overview of the experimental workflow Created with BioRender.com.

***Note:*** The reason for starting the preparatory steps a week before the actual experiment is that passaging HEK293T cells taken from the cryostock at least once allows for similar cell growth rates in all samples. The steps included in this chapter are shown schematically in Figure 1.

3. Transfer the cryovial with the cells from cryostorage with liquid nitrogen to the cell culture room wearing protective cryogenic gloves resistant to low temperatures.
4. Place the cryovial with frozen HEK293T cells in a 37.0°C water bath to thaw.

**CRITICAL:** It is important to thaw the cells quickly e.g. during 2 minutes (min) and minimize the handling time to avoid the toxic effects of the cryoprotectant dimethyl sulfoxide (DMSO).

5. Using 1 mL pipette with a sterile filter tip, add the thawed HEK293T cell suspension to the tissue culture dish 100 (60.1 cm^2^) with 10 mL culture media and grow the cells at 37.0°C in an incubator with 5.0% CO_2_ atmosphere until 80% confluence.
6. Passage HEK293T cells (P. +1) to a new tissue culture dish 100 (60.1 cm^2^) pre-filled with 10 mL of medium and allow the cells to grow until they reach approximately 80% confluence.

***Note:*** After removing the medium during passaging the HEK293T cells, use warm phosphate buffered saline (PBS) to wash away any floating cells and FBS debris and continue with trypsin (0.025% in PBS for 5 min) digestion, which should be stopped by the addition of new media.

**CRITICAL:** After trypsin digestion and adding new media, count the cells, using e.g. Corning Cell Counter (Life Sciences, 6749), according to vendors instructions.

### Plasmid preparation

7. Create or purchase the plasmids for an experiment from a vendor (e.g., Clontech), transform them into bacteria and pre-cultivate. If interested in working with CMV-promoter driven human *ATF4* HA-tag plasmids from the associated primary research manuscript, contact the lead contact.
  a. Incubate the plates with bacteria, containing plasmids, for 16 h in a bacterial incubator at 37°C.
    ***Note:*** Antibiotic-containing (for selection of plasmids with a resistance gene, e.g. Kanamycin/Neomycin) LB plates should be prepared in advance and stored at 4°C.
  b. Inoculate the bacterial cultures from the plates into culture tubes containing LB media with a proper antibiotic and incubate them for 16 h in a shaking incubator at 37°C.
    ***Note:*** Complete details of the *ATF4*-HA plasmids construction and cloning can be found in the study by Smirnova et al.^1^
    ***Optional:*** The current protocol describes the specific steps for using HA tag reporter constructs, but we have also successfully used c-Myc tag constructs in our associated research paper, which are also available upon request.
8. Isolate the plasmids using the QIAGEN Plasmid Mini Kit and QIAGEN Plasmid Purification Handbook using the manufacturer’s instructions: www.qiagen.com/HB-1193.
  a. Elute the DNA of the isolated plasmid in 50 μL of UltraPure Distilled water.
  b. Purify the plasmids using QIAquick PCR Purification Kit to minimize the contribution of the plasmid DNA prep to cellular toxicity during an experiment and to increase the efficiency of human cell culture transfection step.
  c. Measure plasmid concentrations on NanoDrop One.

***Note:*** Dilute the individual isolated plasmids to obtain equal concentrations of all of them; optimal concentration for the experiment was found to be 450–500 ng/μL.

**CRITICAL:** The use of freshly prepared plasmids for each experiment makes the transfection step of this protocol more efficient.

### Preparation of ER stress inducing reagent

9. Order the ER stress inducing reagent thapsigargin (Tg) from the manufacturer and prepare 1 mM stock solution by dissolving the powder in DMSO.

***Note:*** The current protocol in the original experimental design was created to induce the cells with Tg for the purpose of studying *ATF4* translational control under integrated stress (ISR) and unfolded protein (UPR) responses, but other reagents of a similar effect such as tunicamycin can be used as well.^1^

## Key resources table

**Table:** 

REAGENT or RESOURCE	SOURCE	IDENTIFIER
**Antibodies**		

Mouse monoclonal anti-HA tag [HA.C5] 1:50 dilution	Abcam	Cat# ab18181; RRID:AB_444303

**Chemicals, peptides, and recombinant proteins**		

Dimethyl sulfoxide (DMSO)	Sigma	Cat# D2438-5X
Thapsigargin (Tg)	Invitrogen	Cat# T7458
Tunicamycin	Sigma-Aldrich	Cat# T7765-1MG
UltraPure distilled water	Thermo Fisher Scientific	Cat# 109977-035
QIAGEN Plasmid Mini Kit	QIAGEN	Cat#12123
QIAquick PCR Purification Kit	QIAGEN	Cat#28104
TurboFect	Thermo Fisher Scientific	Cat# R0532
1× Glo lysis buffer	Promega	Cat# E2661
RNA blue	Top-Bio	Cat# R013
Chloroform	VWR BDH Chemicals	Cat# 22711.290
2-Propanol	VWR BDH Chemicals	Cat# 20842.312
Ethanol 96% vol	VWR BDH Chemicals	Cat# 20822.290
GlycoBlue Blue Coprecipitant	Thermo Fisher Scientific	Cat# AM9516
TURBO DNA-free Kit	Invitrogen	Cat# AM1907
High-capacity cDNA reverse transcription kit	Applied Biosystems	Cat# 4368814
RNase inhibitor	Applied Biosystems	Cat# N8080119
HOT FIREPol EvaGreen qPCR Mix Plus 5×	Solis BioDyne	Cat# 08-25-00020
Bio-Rad Protein assay dye reagent concentrate	Bio-Rad	Cat# 5000006

**Critical commercial assays**		

12–230 kDa Fluorescence Separation Module	ProteinSimple Bio-Techne	SM-FL004
2–40 kDa Separation Module	ProteinSimple Bio-Techne	SM-0W009
EZ Standard Pack 1	ProteinSimple Bio-Techne	Cat#PS-ST01EZ-8
Anti-Mouse Detection Module	ProteinSimple Bio-Techne	DM-002
Protein Normalization (PN) Module	ProteinSimple Bio-Techne	DM-PN02

**Experimental models: Cell lines**		

HEK293T	ATCC	CRL-3216; RRID: CVCL_0063

**Oligonucleotides**		

h*ATF4*-HA tag-F GAGATCCAGTACCTGAAAGATTTG	Smirnova et al.^1^	N/A
h*ATF4*-HA tag-R GTAATCTGGAACATCGTATGGG	Smirnova et al.^1^	N/A
Neomycin-F GAACTGTTCGCCAGGCTCAAG	Smirnova et al.^1^	N/A
Neomycin-R GGCCACAGTCGATGAATCCAG	Smirnova et al.^1^	N/A
18S-F CCGCAGCTAGGAATAATG	Herrmannová et al.^2^	N/A
18S-R CCGGTCCAAGAATTTCAC	Herrmannová et al.^2^	N/A
yeast RPL41-F CGAAATGAGAGCCAAGTGG	Herrmannová et al.^2^	N/A
yeast RPL41-R ATGCAATTTAGATCCATTATGAGG	Herrmannová et al.^2^	N/A

**Software and algorithms**		

Compass for SW ver. 6.3.0	ProteinSimple	https://www.bio-techne.com/resources/instrument-software-download-center

**Other**		

Jess automated western blot system	ProteinSimple Bio-Techne	004-650
Corning cell counter	Corning Life Sciences	6749
NanoDrop One	Thermo Scientific	ND-ONE-W

## Step-by-step method details

### Sample preparation

**Timing: 2 days**

**Figure 2:**
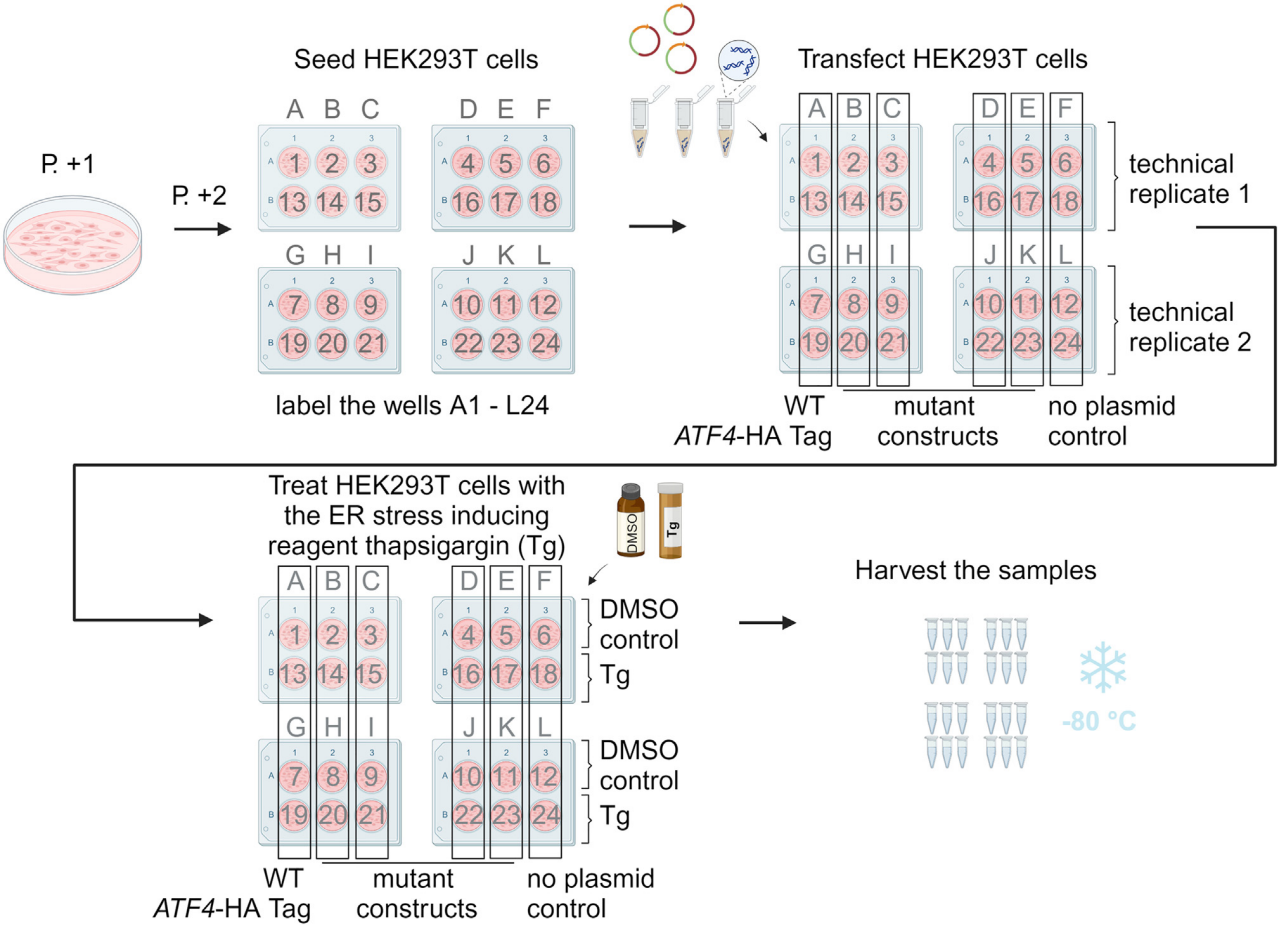
Step-by-step method details: general scheme of sample preparation Created with BioRender.com.

This step describes sample preparation from the pre-grown HEK293T cells transfected with human *ATF4* HA-tag reporter constructs, which is detailed in Figure 2. Approximately 6 × 10^6^ cells *per* well were seeded to obtain 24 samples of cell lysates with a stock sample (total protein) concentration of at least 1.5 mg/mL (explained in detailed in the following section).

1. Start your experiment by seeding HEK293T cells that are pre-grown in a 60.1 cm^2^ tissue culture dish (for details see the previous chapter of the current protocol: before you begin; cells preparation). Seed cells into a six-well plate (Techno Plastic Products) in a number of 2.5 × 10^5^ cells into 2.5 mL of medium *per* well, for a total of 24 wells.

***Note:*** To passage the cells, use the same procedure as described in the before you begin. Cells preparation chapter, including the steps for sterilizing the work area, the medium prewarming, PBS wash, trypsin digestion and counting the cells.

2. Twenty-four hours after seeding, transfect HEK293T cells (50–70% confluency) with the prepared plasmids. Prepare 12 × reactions (for two wells in pairs, e.g., 1 + 13, 2 + 14, 3 + 15, 4 + 16, etc. in Figure 2) using the following scheme:
  a. To 12 × 1.5 mL microcentrifuge tubes add 1 mL of serum-free DMEM (no FBS).
  b. Add 5 μg of plasmid DNA to each tube with medium (i.e., 2.5 μg per well).
  c. Vortex the tubes.
  d. Add 10 μL of TurboFect transfection reagent (Thermo Fisher) to each reaction.
    ***Note:*** the protocol for using the TurboFect provided by the supplier is available here: https://www.thermofisher.com/document-connect/document-connect.html?url=https://assets.thermofisher.com/TFS-Assets%2FLSG%2Fmanuals%2FMAN0013147_TurboFect_Transfection_Reagent_UG.pdf
  e. Vortex the tubes and let them stand at 23°C for 20 min.
  f. Add 0.5 mL of the reaction dropwise into each well (one reaction *per* two paired wells).
    ***Note:*** Positive control (WT construct) and negative control (no plasmid) should be included for all experiments.
    ***Optional:*** Including two technical replicates in an experiment will increase the reliability, accuracy, and therefore the consistency of the results. For technical replicates, the transfection reaction for the same plasmid can be prepared as one Master Mix. We opted for two separate transfection reactions but we still consider those as technical not biological replicates as described in Figure 2.
3. Eight hours after transfection, treat half of the cells with pre-dissolved ER stress inducing reagent (Tg stock concentration 1 mM, final concentration 1 μM) and the other half with the DMSO control. Prepare Master Mix for 12 + 1 extra well.
  a. Prepare two 15 mL centrifuge tubes with 6.5 mL of DMEM with FBS, i.e., 0.5 mL *per* well.
  b. To one tube add 45.5 μL of 1 mM Tg stock, mix by vortexing.
  c. To the other tube add 45.5 μL of DMSO, mix by vortexing.
  d. Add 0.5 mL of Tg or DMSO Master Mix dropwise into each well in accordance with the suggested scheme in Figure 2.

***Note:*** The current protocol in the original experiment design is set for three hours of Tg treatment, but treatment with 0.5 μM tunicamycin for four hours has been successfully used in the associated primary research manuscript.

4. Incubate the cells for 3 h with the Tg/DMSO reagents and then harvest the cells:
  a. Aspirate the media from three wells in the first row of the first six-well plate and add 200 μL of standard 1× Glo Lysis Buffer supplemented with protease inhibitors by the vendor (Promega), pre-warmed at 23°C 30 min before use, per well.
    i. Repeat the same procedure in the second row of the six-well plate.
    ii. Follow the same procedure for the other plates.
      ***Note:*** Aspirate the media carefully so that it is not diluting your final lysate. Aspirating media from only 3 wells at a time minimizes the time when the cells are drying out.
  b. Incubate the six-well plates in a Eppendorf Thermomixer Comfort 5355 Block MTP for 5 min at 550 rpm at 25°C.
  c. Collect the cell lysate from each well (A1-L24, Figure 2) into pre-labeled and pre-chilled microcentrifuge tubes on ice.
    ***Note:*** Collect the lysate using a 1 mL filter tip and try to avoid bubbles.
  d. Vortex and spin all the samples in pre-cooled centrifuge for 5 min at 16,000 × *g* at 4°C.
  e. Transfer 180 μL of the supernatant into new tube on ice.
    i. Prepare 4 × 20 μL aliquots of the cell lysate for the Jess assay and flash freeze the samples in liquid nitrogen.
    ii. Transfer 90 μL of the cell lysate to pre-chilled 1.5 mL RNase/DNase free microcentrifuge tubes and add 750 μL of RNA Blue reagent (Top-Bio, Trizol alternative).
      ***Note:*** These samples will be used for the following RNA isolation and RT-qPCR mRNA levels control steps.
  f. Store all samples at −80°C.
    **Pause point:** The samples can be stored at −80°C for at least several months.

### Equal mRNA levels control step

**Timing: 1 day**

The following steps are important control steps to ensure that the mRNA levels of the transfected constructs are equal. Only samples that pass this control step will be selected for the Jess assay. The readers are welcome to perform the total RNA isolation and qPCR using standard protocol established in their labs. Here we provide an example of our procedure that combines several standard protocols from different vendors.

5. Isolate total RNA from the samples in RNA Blue (Top-bio) stored at −80°C.

***Note:*** Top-bio manufacturer’s protocol is available here: https://www.top-bio.com/img/uploaded/639_PLen-RNA-Blue-rev-012022.pdf

***Optional:*** Using GlycoBlue Coprecipitant during the total RNA isolation will help to better visualize the RNA pellets.

6. Perform a digestion step with the TURBO DNA-free Kit (Invitrogen).

***Note:*** Invitrogen TURBO DNA-free Kit protocol is available online: https://www.thermofisher.com/document-connect/document-connect.html?url=https://assets.thermofisher.com/TFS-Assets%2FLSG%2Fmanuals%2F1907M_turbodnafree_UG.pdf

***Optional:*** For the DNase treatment, it is possible to use 0.8 U (units) of the TURBO DNase Enzyme for 20 μL RNA samples with concentrations in the range of 1–5 μg (measured with, for example, NanoDrop) and then incubate the samples for one hour at 37°C followed by termination of the reaction with 2 μL of DNase Inactivation Reagent.

7. Use equal amounts of total RNA, e.g., 0.5 μg per each sample, to synthesize cDNA using High-capacity cDNA reverse Transcription Kit and RNase Inhibitor (Applied Biosystems). Incubate the reactions for 10 min at 25°C, followed by 120 min at 37°C and 5 min at 85°C, with a subsequent temperature drop to 4°C to allow the samples to be used for the next steps.

***Note:*** Instructions on the usage of High-capacity cDNA reverse Transcription Kit are here: https://www.thermofisher.com/document-connect/document-connect.html?url=https://assets.thermofisher.com/TFS-Assets%2FLSG%2Fmanuals%2FMAN0017977_highcap_cDNA_RT_UG.pdf

***Optional:*** Add spike RNA to 20 μL of each reaction before cDNA synthesis to control the efficiency of the cDNA synthesis step. We routinely use for this purpose 1 μL of yeast *S. cerevisiae* RPL41 RNA with a concentration in the range of 0.5–1 ng/μL.

8. For 10 μL qPCR reactions, mix HOT FIREPol EvaGreen qPCR Mix Plus, 5× (Solis BioDyne) (data sheet is available here: https://solisbiodyne.com/pics/9800_DS-08-25_v3_HOT_FIREPol_EvaGreen_qPCR_Mix_Plus_no_ROX_revised_12.04.2022.pdf), cDNA and 0.03 μL of 300 nM forward primer and 0.03 μL of 300 nM reverse primer (either those described in the associated research paper or in the current protocol in key recourses table (KRT)). Run qPCR using an appropriate program, an example of which can be found in Table 1.

**Figure 3:**
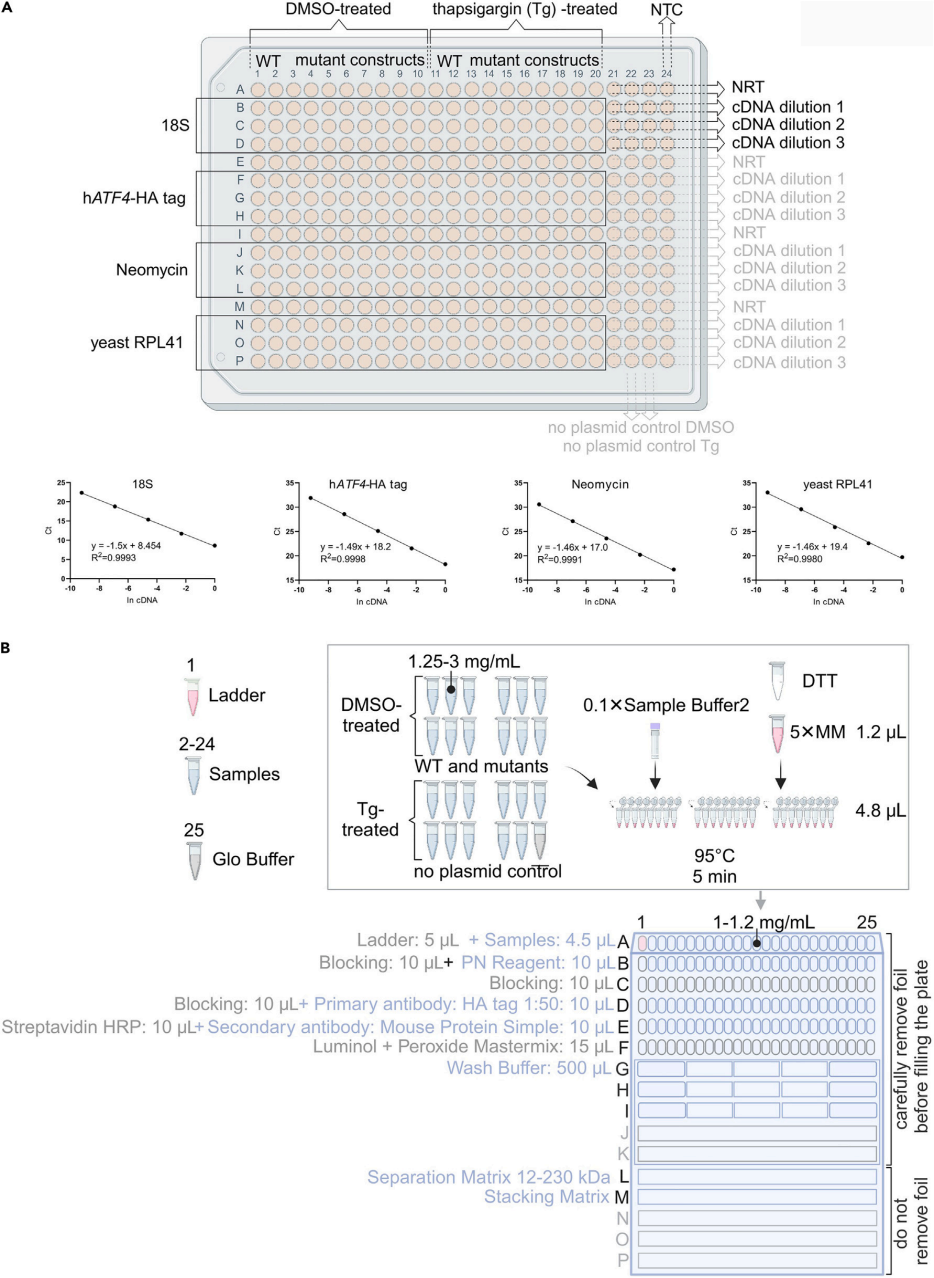
Step-by-step method details (A) Upper part – scheme of qPCR plate for mRNA levels control; bottom part – estimated primer efficiency for each primer pair. (B) Upper part – Jess assay experimental workflow; bottom part – Jess plate with suggested scheme for pipetting of the reagents. Created with BioRender.com.

***Optional:*** Using the Bio-Rad CFX384 Real-Time PCR system will allow for multiple constructs to be efficiently validated and compared in a single qPCR run; an example of such a qPCR plate is shown in Figure 3A (upper part).

***Note:*** To compare *ATF4*-HA expression levels between WT and mutant constructs, a reverse primer matching the HA tag should be used to eliminate interference by the endogenous *ATF4* mRNA.

**CRITICAL:** To control for the same transfection efficiency, primers corresponding to the plasmid region encoding neomycin/kanamycin resistance should be included for each pair of constructs being compared in each experiment, as well as controls for RNA isolation (18S rRNA primers) and cDNA synthesis efficiency (yeast RPL41 primers), as summarized in KRT. Primer efficiency has to be tested prior to the actual Real-Time PCR experiment. The PCR efficiency has to be determined for both the h*ATF4*-HA tag and the controls, the threshold crossing point (Ct) values must be linearly correlated with the logarithmic value of the cDNA amount (ln cDNA) for the slope of this line to inform the PCR efficiency at the given parameters. An example of a representative experiment for each primer pair used in this protocol (h*ATF4*-HA tag, Neomycin, 18S, yeast RPL41) is shown in Figure 3A (bottom part), where the template was used in several dilutions for reactions (ln cDNA) and the obtained Ct values were plotted to a graph. Real-time PCR efficiency was estimated for 18S primers to be 1.95, hATF4-HA tag 1.96, Neomycin 1.98, and yeast RPL41 1.98, and was calculated according to.^3^

9. Analyze results using Bio-Rad CFX Manager.

***Note:*** At a minimum, three 10-fold serial dilutions of cDNA should be tested for each construct to be compared to WT, and the maximum difference criterion of 1 cycle must be met for all three sample dilutions in all corresponding controls, or else samples must be discarded from further analysis.

**Pause point:** The cDNA samples can be stored at −20°C for at least one week.

**Table 1:** Example of qPCR program that can be used for validation and mRNA level comparison between the constructs

Step	Temperature	Time
1	95°C	15:00
2	95°C	0:15
3	60°C	0:20
4	72°C	0:20
5	Plateread	
6	Go to step 2, 43× more times	
7	72°C	3:00
8	65°C	0:31
9	65°C, 0:05 + 0.5°C/ cycle, Ramp 0.5°C/s	
10	Plateread	
11	Go to step 9, 50× more times

### The Jess assay

**Timing: 1 day**

These steps describe detection and quantification of the ATF4-HA tag protein in the samples using Jess assay exclusively in samples that met the maximum difference criterion in the previous Equal mRNA levels control step (the criterion represents difference within 1 cycle between WT and mutant *ATF4-HA* sample in qPCR). Complete details can be found in STAR Methods in Smirnova et al.^1^

10. For all samples that passed the 1 cycle criterion, run the Jess Simple western (SW) assay following the manufacturer’s instruction obtained when the instrument was installed, with the following exceptions and modifications. An example of a typical Jess machine run is outlined in Table 2.
11. Make the deep-frozen aliquots of cell lysates from the previous sample preparation step (4.e.i.) ready for the Jess assay:
  a. Thaw one aliquot of cell lysates for each technical replicate of the DMSO control and Tg-treated WT and mutant samples, as well as for no plasmid control, example is shown in Figure 3B (upper part).
    **CRITICAL:** The number of samples that can be included in each run is 24 plus one extra capillary for the ladder, and the manufacturer’s requirement is that the full set of capillaries must always be run for the sake of precision of the entire analysis, therefore, if less than 24 capillaries are planned to be occupied, 1× Glo Lysis buffer can be used instead of a missing sample(s) to avoid empty capillaries.
  b. Measure the total protein concentration in each sample using the Bio-Rad Protein Assay.
    ***Note:*** Performing the BSA (Bovine serum albumin) titration curve with serial dilutions of BSA in PBS is highly recommended before each run.
  c. Adjust the total protein concentration in all samples to the same value with the 1× Glo Lysis buffer.
    ***Note:*** The sample stock concentration should be between 1.5-3 mg/mL of total protein.
  d. Mix the cell lysates with 1× Sample Buffer 2 (Protein Simple Bio-techne) so that the target sample concentration in a well is 1.2 mg/mL.
    ***Note:*** For example, sample stock concentration = 3 mg/mL; Target sample concentration in well = 1.2 mg/mL; mix 2.4 μL sample with 2.4 μL 1× Sample Buffer 2; total sample volume will be 6 μL – see step e.
    ***Optional:*** For dilutions, it is more convenient to use PCR tube strips with attached flat caps instead of conventional microcentrifuge tubes, as shown in Figure 3B (upper part).
    **CRITICAL:** For the Protein Normalization (PN) kit, the target sample concentration in the well of the Jess plate must be in the range 1–1.2 mg/mL for each sample (1.2 mg/mL is recommended), and the maximum volume of each sample diluted in the buffer is 4.8 μL per well.
  e. Using a Repetman (Gilson), add 1.2 μL of the reconstituted 5× Master Mix containing DTT solution (Protein Simple Bio-techne) to each 4.8 μL of pre-diluted sample to obtain a final sample volume of 6 μL (25% more than needed for loading in a plate well).
    i. Gently mix all samples (in strips or tubes) by vortexing.
    ii. Spin for 30 s (sec) at 1,150 × *g* using e.g., Bio Multi-spin benchtop microcentrifuge (Biosan) to collect the sample at the bottom of the tube.
      ***Note:*** It is important to carefully and fully dissolve the lyophilized contents of the commercial tubes for 5x Master Mix. The tubes contain fluorescent standards that are crucial for assigning the correct molecular weight (MW) of the protein of interest.
  f. Incubate samples at 95°C for 5 min in thermomixer with adapter for PCR tubes or PCR cycler.
    **CRITICAL:** While pipetting 5× Master Mix to samples, it is important to close the tubes one by one after adding the Master Mix to prevent evaporation of small sample volumes.
  g. After denaturation, place samples on ice for 1 min, then vortex and spin, as in the preceding step, to make the samples ready to be loaded into the plate wells.
12. Remove the protective film from 12-230 kDa Pre-filled Plate from Fluorescence Separation Module (Protein Simple Bio-techne).
  a. Pipette in the first well (A1) 5 μL of the reconstituted Biotinylated Ladder (Protein Simple Bio-techne).
  b. Pipette 4.5 μL of the experimental samples 1–24 to wells A2-A25 as shown in Figure 3B (bottom part).

***Note:*** A control with 1× Glo Lysis buffer only (no lysate) is recommended to be included in the first Jess test run. Troubleshooting 1.

13. Pipette the reagents (Protein Simple Bio-techne) into the Plate according to the scheme in Figure 3B (bottom part).
  a. Pipette 10 μL of the Antibody diluent 2, which is provided by Protein Simple bio-techne as a part of the Anti-Mouse Detection Module and included in key resources table (KRT), into B1, C1-C25 and D1 wells using a Repetman.
    **CRITICAL:** B2-B25 should be left empty for the later addition of the PN reagent (hereinafter in the protocol).
  b. Pipette 10 μL of the Primary antibody at the required concentration in the Antibody Diluent 2 (refer to a previous point a.) into wells D2-D25. For anti-HA tag (abcam) antibody used in the current protocol we recommend 1:50 dilution.
    ***Optional:*** The current protocol and the associated primary manuscript specify the use of mouse monoclonal anti-HA tag antibody (abcam) at the dilution ratio of 1:50 in the Antibody Diluent 2, but other antibodies may be used and should be tested prior to the actual Jess experiment. Troubleshooting 2.
    ***Note:*** For a pilot run of the antibody with the sample in Jess, a single concentration can be chosen, for example 10× higher than the one recommended for use in a classical western blotting experiment. High quality, e.g. Co-IP-grade antibodies, should work well in the Jess assay with a higher probability.
    **CRITICAL:** It is crucial to perform an antibody titration for each newly supplied antibody vial to find the optimum dilution (concentration) at which the saturation occurs, otherwise the assay performance may fluctuate. Troubleshooting 3 and 4.
  c. Pipette 10 μL of Streptavidin-HRP in well E1.
    **CRITICAL:** Always double check the expiration date of all reagents to ensure that the Jess assay works properly.
  d. Pipette 10 μL of the secondary antibody from the Anti-Mouse Detection Module (Protein Simple Bio-techne) in the wells E2-25 using a Repetman.
    ***Optional:*** It is also possible to use Anti-Rabbit Detection Module (Protein Simple Bio-techne), depending on the design of the assay and the primary antibody choice.
  e. Mix 200 μL of Luminol (Protein Simple Bio-techne) and 200 μL of Peroxide (Protein Simple Bio-techne) in a microcentrifuge tube and pipette 15 μL in wells F1-25 using a Repetman.
    ***Note:*** Keep Protein Simple Bio-techne stock reagents and the diluted primary antibodies on ice during the pipetting and return them to 4°C immediately after use.
    **CRITICAL:** During preparation of the reagents or pauses in pipetting, cover the plate with its lid to prevent reagents from evaporating.
14. Recover the PN reagent from −80°C and let it warm up on ice for 2 min:
  a. Add 100 μL of the Reconstitution Reagent and dissolve PN reagent by pipetting.
  b. Transfer 50 μL of the dissolved PN reagent to new microcentrifuge tube.
  c. Further, dilute it by adding 250 μL of the Reconstitution Reagent (the final volume is now 300 μL).

***Note:*** Once reconstituted, keep the PN reagent on the bench at 23°C.

15. Centrifuge the plate at 1,000 ×*g* for 5 min at RT and then use a 10 μL pipette to remove bubbles if present at the bottom of the plate wells.

***Note:*** Always make sure to centrifuge the plate at RT (never at 4°C), centrifugation of the prefilled plate at 4°C may influence the assay result.

**CRITICAL:** Remove the bubbles at the bottom of the wells in the first row with the ladder and samples, since they can affect the overall outcome of the assay when the ladder and/or samples get aspirated.

16. Add 500 μL of the wash buffer in rows G, H and I, and carefully remove the remaining foil from the evaporation sensitive part of the plate: rows L-P.

***Note:*** Remove any bubbles that might appear during the foil removal.

17. Carefully transfer the plate, covered with the lid, to the Jess instrument and get ready to start the Jess run.
  a. Switch on the instrument and the PC. Open the door of the Jess machine and wipe it gently with 70% isopropyl alcohol to clear away the dust and to prevent background fluorescence signal detection during capturing.
  b. Create a workflow in Jess.
    i. Open the Simple Western instrument software: Compass for Simple Western (SW) software.
    ii. At the top right corner, select New Assay to set it up.
      ***Optional:*** It is possible to open a script from earlier runs and modify it for a new use.
    iii. Fill in information about the Jess run, according to the plate design with the reagents that were pre-loaded in the previous steps.
      ***Optional:*** Save the Template before running the Jess assay and then use it for other ongoing/planned experiments.
    iv. Choose the required settings in the Compass for SW software. Use the example of the Jess protocol in Table 2.
  c. Remove the plate lid and insert the plate into the instrument.
  d. Carefully open the capillary box and quickly insert the capillary cartridge into the cartridge holder to minimize capillaries’ exposure to light.
    ***Note:*** The blue light inside the instrument indicates the correct placement of the inserted capillaries.
    **CRITICAL:** Do not touch the capillaries during the manipulation.
  e. Close the Jess instrument door and wait for the red light on the door to change to blue, then initiate the instrument run.
    i. Press the Connect button.
    ii. Select the Start button.
    iii. Wait until the cartridge is detected – the corresponding message should appear on the display.
    iv. Confirm that the plate is filled with 15 μL of the Luminol and Peroxide in each well. Troubleshooting 5.
      ***Optional:*** Run Jess self-test prior to the first run and after every 3–4 runs thereafter.
      **CRITICAL:** The Jess machine should be kept in a room where the temperature is maintained between 18°C-23°C, as any other temperature may affect the migration of the samples in the matrix and thus the assay’s outcome.
      ***Optional:*** Wait for the Jess machine to display the running timeline. A few min after the launch, check if the CCD camera image with the detected capillary has appeared.
18. Wait until the run is done and remove the plate as well as capillaries from the machine and then switch it off. The completed run is now ready for its analysis.

***Note:*** An overview of the individual steps of the Jess machine run and the general timetable is shown in Table 3.

**Pause point:** The sample aliquots for repeating Jess run, if it is found to be necessary, can be stored at −80°C for at least several months.

**Table 2:** Example of Jess protocol

	Value
**Separation Matrix**	

Well Row	L1
Load Time (sec)	200.0

**Stacking Matrix**	

Well Row	M1
Load Time (sec)	12.0

**Sample**	

Well Row	A1
Load Time (sec)	6.0
Separation Time (min)	30.0
Separation Voltage (volts)	375
Standards Exposure (sec)	4.0
EE Immobilization Time (sec)	200.0
Protein Normalization (PN) Time (min)	25.0
Well Row	B1
Antibody Diluent Time (min)	5.0
Well Row	C1
Primary Antibody Time (min)	30.0
Well Row	D1
Secondary Antibody Time (min)	30.0
Well Row	E1

**Detection**	

Well Row	F1
Detection Profile (Chemi)	HDR
Detection Profile (NIR)	None
Detection Profile (IR)	None
Ladder Channel	CHEMI

**Table 3:** Jess machine run step-by-step overview

Step	Description
1	Separation and stacking matrixes are pre-aspirated in a capillary
2	Proteins in a capillary are separated according to their MW
3	Proteins are immobilized by UV light to a capillary wall
4	Clearing of the matrix from the capillary step
5	Initiation of immunoprobing process: incubation with primary antibody, secondary HRP conjugate and chemiluminescent substrate
6	The emitted chemiluminescent light is recorded by CCD camera and then quantified

### Data analysis

**Timing: 1 day**

The following analysis can be performed on any PC independently of the instrument supplied by the manufacturer. The Simple Western instrument software Compass for SW is available online at https://www.bio-techne.com/resources/instrument-software-download-center?filters%5Binstrument_category%5D%5B0%5D=372 . The following data analysis chapter of the current protocol presents an overview of the general analysis created to provide an introduction into the procedure. In addition, it contains useful tips that we have discovered while conducting the experiments and during the troubleshooting communication with the Jess Protein Simple Bio-techne staff. More detailed instructions are provided by the vendor when the instrument is installed in the laboratory.

**CRITICAL:** Before running the Jess experiment or even the self-test, double check that the latest release of the Compass for SW software is installed on both the instrument and the PC on which the analysis is to be performed.

19. Open the Jess run in the Compass SW software. Begin the data analysis with checking the Run summary:
  a. Carefully check the separation video recorded by the CCD camera.
  b. Check the IV Plot.

***Note:*** Play the Separation run summary and keep track of bubbles in the capillaries during the sample separation. In case bubbles appear, make a note of it or directly discard the affected sample from the analysis.

20. Continue by selecting the Analysis menu at the top right corner, starting with Standards.

***Note:*** There is a 5× Master Mix in each sample which contains fluorescent standards (Std) that appear as peaks with the defined MW: Std1, Std 29 and Std 230. Troubleshooting 6.

21. At the top left corner, select Samples from the menu bar. At first, check that the Ladder (“Ldr 12–230” is the shortening for Ladder in commercial Compass for SW software) peaks and samples look as expected:
  a. Confirm that six peaks appear in the Ladder MW (kDa): Ldr 12, Ldr 40, Ldr 66, Ldr 116, Ldr 180 and Ldr 230. Troubleshooting 7.
    ***Note:*** The Ladder is always displayed in capillary 1.
  b. Check the samples 1–24 in the capillaries 2–25 in the chemiluminescence channel and make sure that only a single peak appears where expected (unless the antibody of choice is known to generate cross-reactions).
    ***Note:*** For details on how the 53 kDa ATF4-HA target peak, displayed as an electropherogram, was defined and validated by western blotting please see Smirnova et al.^1^
    **CRITICAL:** The optimal range of the chemiluminescent signal is 3,000 to 90,000 U. Troubleshooting 8.
    ***Optional:*** The current protocol uses the 12–230 kDa Fluorescence Separation Module. For proteins that are smaller in size, it is recommended to use a different 2–40 kDa Separation Module SM-0W009 (Protein Simple Bio-techne).
  c. Check across samples in the Graph options menu bar for Baseline Fit (good antibodies should have low baseline noise) and All Exposures view for all samples.
    ***Note:*** All Exposures can be zoomed in for better viewing and the five shortest exposures should be clustered together. Troubleshooting 9.
  d. Overlay the chemiluminescence channel with the fluorescent PN channel and check it for all the samples. In the Capillaries and corrected area (Corr. Area) section, find PN Corr. Area, which should be greater than 22,000 U (the upper limit can be e.g., 45,000 U ), otherwise discard the samples from the analysis. Troubleshooting 10.
    ***Note:*** By default, the region used for normalization is set to 12–220 kDa. This can be changed in Edit/Analysis/Normalization menu. We recommend to set the Area End (kDa) value to 180 as advised by the Protein Simple Bio-techne staff. The reference capillary for the following constructs comparison can be found and changed in the same tab. Troubleshooting 11, 12, and 13.
22. Analyze samples and make comparisons between the WT and mutant constructs.
  a. Return to the Chemiluminescence channel. Select the peak of interest and Name peak.
    ***Note:*** If the software does not automatically recognize the target peak, select Add Peak.
    ***Optional:*** Choose Selected if you need to name a peak only in a specific capillary or select All to name the peaks in all samples.
  b. In the capillaries to be compared, check Peak Fit, which can be highlighted using the menu bar at the upper right corner. For more details on how the ATF4-HA target peak area at 53 kDa should be determined, please refer to Smirnova et al.^1^
    ***Note:*** The green area under the target peak should automatically match the black border of the curve, if it does not, adjust it manually by changing the target Peak fit or by rearranging the surrounding peaks and selecting Add peak.
    ***Optional:*** In case the peak still does not fit correctly, it is also possible to change the Peak Fit at the top left menu bar Analysis: Peak Fit. The parameters Threshold and Width are available in the Peak Fit, and the automatically set Threshold 10 can be changed to 9.0 or 8.0, while Width 9.0 can be changed to 8.0 or 7.0. Note that any other manipulations with these parameters should be avoided.
  c. To compare WT and mutant samples, select all capillaries to be analyzed and turn on the fluorescent PN channel to check that loading of the selected samples is equal. Next to the PN Corr. Area, the PN difference in percentage is displayed, set the WT sample as a reference capillary – it would be shown as the 100% PN loading.
    **CRITICAL:** If the difference in the PN loading between the WT and mutant samples is greater than 20% (i.e. more than 120% or less than 80%), discard these samples from your analysis.
  d. In the Capillaries menu under the peak electropherogram, the corrected values after automatic normalization of the PN loading to the reference capillary for the target peak are shown in the named peak section.
    ***Note:*** These are the final values that can be used to compare differences in protein expression levels between your WT and mutant constructs.

## Expected outcomes

This protocol describes preparation of the samples from the HEK293T cells line transfected with human *ATF4* HA-tag reported constructs with their subsequent detection in the Jess assay. This chapter includes data from the sample detection that successfully passed all the control steps described above, namely in the sections before you begin, sample preparation and equal mRNA levels control step.

**Figure 4:**
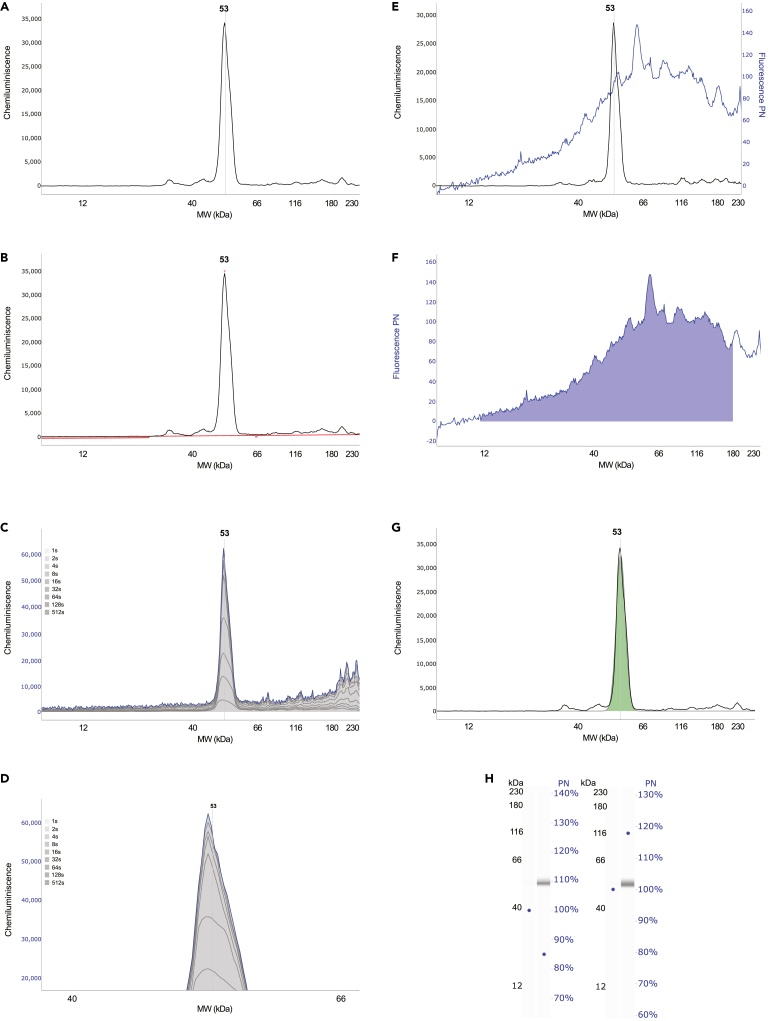
Expected outcomes (A) Electropherogram of WT ATF4-HA. (B) Example of good baseline fit. (C) All Exposures view. (D) Zoomed in All Exposures view (E) Overlay of Fluorescent and Chemiluminescent channels (F) PN area (G) Example of good peak fit (H) Example of acceptable protein loading difference.

First of all, in order to be able to detect the appropriate signal and to investigate the differences between different human *ATF4*-HA reporter constructs in Jess machine, HEK293T cells transfected with human *ATF4-*HA plasmids and treated with Tg should show a strong ATF4-HA signal under stress conditions. A single peak with a distinguishable size of 53 kDa can be displayed as an electropherogram in the graph view in Compass SW software. The example of correct detection is shown for WT ATF4-HA tag construct (Tg stress conditions) in Figure 4A. All the necessary verification steps to show that the 53 kDa peak is the protein of interest in our Jess assay are provided in the related research manuscript.^1^

The expected chemiluminescence U that can be obtained using anti-HA tag (abcam) antibodies are approximately 30,000 U.

Next, quality control step of the signal detected using anti-HA antibody has to be performed. The case, when baseline fit of the anti-HA antibody used to detect the target protein is good and the background noise is low, is shown in Figure 4B (in red color).

The shortest exposures (i.e., 1s, 2s, 4s, 8s and 16s) in the “All Exposures” view are spaced equidistant from each other by a small distance (Figure 4C), which is also clearly visible in the zoomed-in version of “All Exposures” (Figure 4D).

And last but not least, careful protein loading control and analysis of the WT and mutants peaks has to be carried out. Correctly loaded samples typically showing values of around 150 U in the fluorescence channel with a PN curve generated automatically in the software are displayed in Figure 4E (in blue).

The area under the PN curve that is automatically calculated when the Jess run is done has borders 12–220 kDa, but the recommended area for use should be changed manually to 12–180 kDa (Figure 4F). The PN curve is auto-quantified, and the resulting values must fall within the range of 22,000–45,000. These final values can be found in the Jess run table given below the electropherogram with the created peak in the PN Corr. Area category.

Once the ATF4-HA peak under Tg stress conditions is correctly detected by the software, the green area inside the peak boundary (Figure 4G) almost perfectly matches the peak curve and is automatically calculated and displayed as the final expression level number of the construct being measured.

The results of the Jess run from the WT and mutant *ATF4*-HA tag constructs can be directly compared due to the same PN loading. An acceptable difference between samples is up to 20%, i.e., within 80% (Figure 4H, the left lane view) or 120% (Figure 4H, the right lane view).

## Limitations

The current protocol is set up for the human *ATF4*-HA reporter system and the signal detection in Jess is accomplished using anti-HA tag antibody (abcam). In the case that the studied mutations would be introduced into the endogenous *ATF4* locus of a cell line using the CRISPR/Cas9 system, antibodies directed against endogenous ATF4 must be used to test the expected effects of the obtained mutants.

One of the major limitations is the batch-to-batch effect of antibodies; therefore, we recommend to titrate each new vial of antibodies.

Another limitation is the relatively high concentration of total protein required for the stock sample to be loaded into each Jess run, i.e., approximately 1.5–3 mg/mL before dilution with 1× Sample Buffer 2. This concentration is required for the proper function of the fluorescent PN normalization module. If achieving this concentration of the sample is not possible, using the RePlex module (RP-001, Bio-techne) in combination with Total Protein Detection Module for Chemiluminescence based total protein assays (DM-TP01) can be tested. As the chemiluminescent detection is more sensitive, this assay can be used with low sample concentrations 0.005–0.2 mg/mL. However, this assay must be first properly optimized to determine that both the antibody and the total protein are within their dynamic range and the chemiluminescent signal from the antibody is not too strong (burning out).

Finally, the Jess assay is a size-based analysis. For a charge-based assay, a different instrument called Peggy Sue (Bio-techne, 004–800) is required and the protocol needs to be optimized separately.

## Troubleshooting

### Problem 1

How to select critical controls for the human *ATF4*-HA protein detection in Jess.

### Potential solution

**Figure 5:**
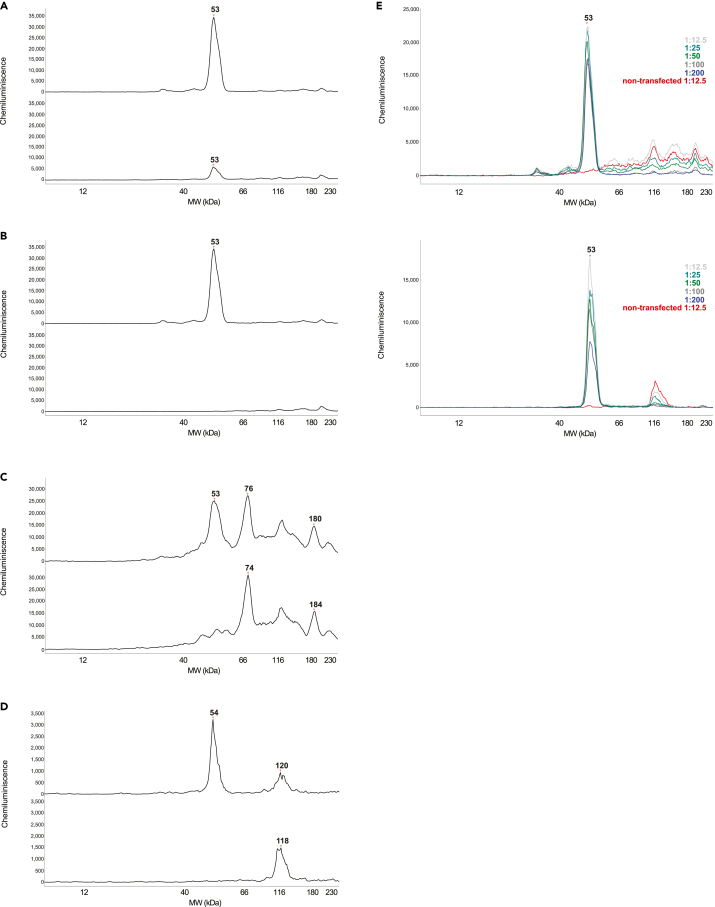
Troubleshooting How to select the proper controls, anti-HA tag antibodies and titrate them for the direct use in Jess assay. (A) WT ATF4-HA construct in stress (upper panel) and non-stress (bottom panel) condition. (B) WT ATF4-HA construct in stress (upper panel) vs. non-transfected control (bottom panel). (C and D) Example of non-optimal antibody signal. (E) Example of antibody titration. Good antibody that reaches saturation (upper panel) and non-optimal antibody (bottom panel).

In addition to the WT Tg stress sample and WT sample in non-stress conditions (Figure 5A) run a non-transfected control (Figure 5B) or no lysate control (1× Glo Lysis Buffer instead of a sample) to verify that the HA signal is present only in the transfected samples (The Jess assay step 12).

### Problem 2

How to choose working anti-HA tag antibodies for the Jess assay (The Jess assay step 13b).

### Potential solution

Test several antibodies from different vendors and select those anti-HA tag antibodies that give a clear single peak (Expected outcomes Figure 4A); i.e., avoid using antibodies giving several non-specific peaks. An example of antibodies raised against HA-tag that we tested in addition to the abcam antibody are shown in Figure 5C (Roche, rat monoclonal, 11867423001) and Figure 5D (Cell Signaling, rabbit monoclonal, 3724).

### Problem 3

How to titrate the anti-HA tag antibodies for the Jess assay (The Jess assay CRITICAL step 13b).

### Potential solution

Test at least five different dilutions of the anti-HA antibody in the Antibody Diluent 2, e.g., 1:500, 1:200 or 1:100, 1:50, 1:25 and 1:12.5 (respectively, 1×, 2.5× or 5×, 10×, 20× and 40× of antibody dilutions that are used for western blotting) (Figure 5E). If you are to decide between several antibodies against the same target, pick those that reach saturating conditions at the highest dilution. A good example is given in the upper electropherogram in Figure 5E, where, with increasing antibody concentration, the peak does not exceed 1.2 × difference in chemiluminescence U compared to another antibody given in the lower electropherogram.

### Problem 4

The antibody titration is done but the peak height of the protein of interest varies from run to run.

### Potential solution

Vortex the antibody thoroughly before pipetting (The Jess assay step 13b).

### Problem 5

When initiating a run, Jess displays an error and does not display the run in progress (The Jess assay step 17).

### Potential solution

Rename the run file to a shorter form.

### Problem 6

**Figure 6:**
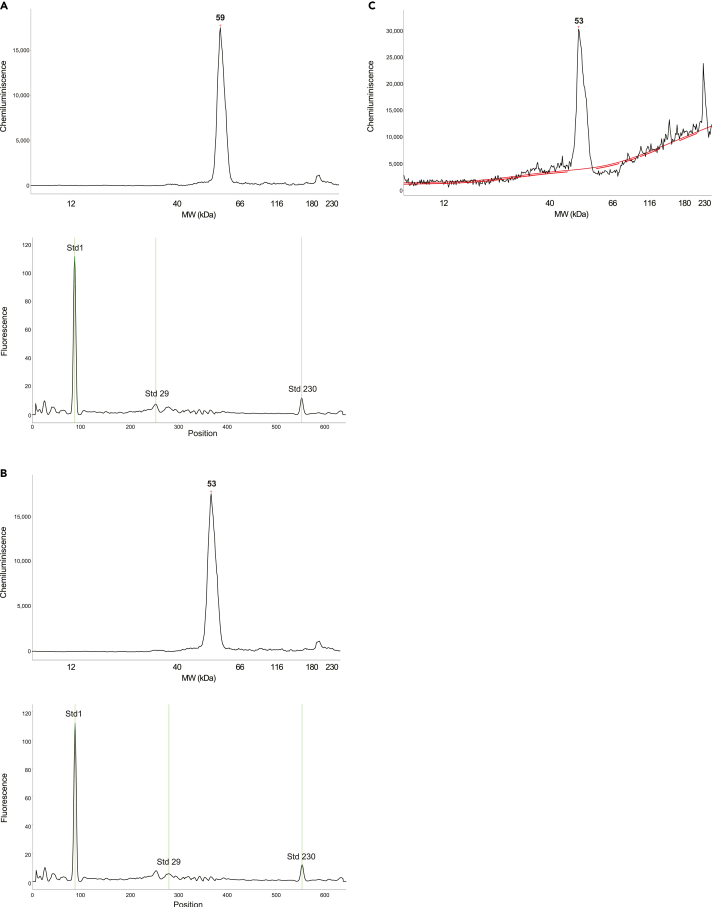
Troubleshooting Errors in the Ladder sizes detection in samples with the target *ATF4*-HA tag protein and bad baseline example for target antibody. (A and B) Example of improper recognition of standard peaks by Compass for SW software and their correction. (C) Example of bad baseline fit with high background noise.

The software incorrectly detects the target peak size (Figure 6A). The standard(s) in the capillary with the ladder look poor (Data analysis step 20).

### Potential solution

In the Standards menu bar for the automatically detected standard peak, set the peak as “Not a Standard”. The peak size should change to the correct position (Figure 6B).

### Problem 7

The peaks of the standards in the capillary are not well-defined (Data analysis step 21a).

### Potential solution

Repeat the entire Jess run.

### Problem 8

The chemiluminescent signal exceeds 90,000 chemiluminescent U and the signal is degrading as judged from the “All exposures” view. Titration of the antibody was performed, and the lowest saturating concentration of antibody is already used (Data analysis step 21b).

### Potential solution

Try using lower sample concentration. However, for optimal performance of the fluorescent PN total protein module (DM-PN02), target sample concentration of 1.2 mg/mL of total protein in a well is recommended. If the chosen antibody gives a too high signal at this sample concentration, chemiluminescent Total Protein Detection Module (DM-TP01) in combination with the RePlex module (RP-001) can be used. For this set up, lower sample concentrations (in range 0.005–0.2 mg/mL of total protein) could be used which should help to get the antibody signal into the optimal range. However, it should be tested prior to experiment that both the antibody signal and the total protein signal are in the linear dynamic range.

### Problem 9

Selected antibody has an inadequate baseline fit and a high baseline noise (Figure 6C) (Data analysis step 21c).

### Potential solution

Perform new titration of the selected antibody in order to optimize its dilution or choose a different antibody.

### Problem 10

**Figure 7:**
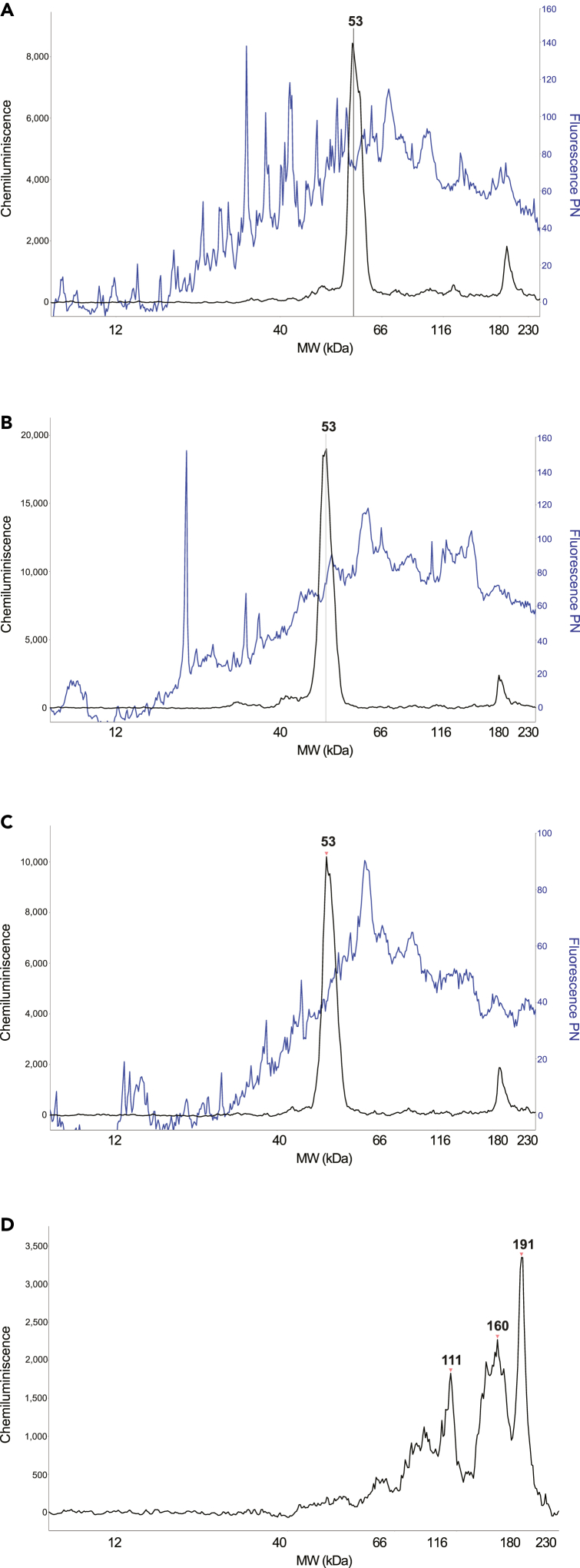
Troubleshooting (A–C) Common errors in fluorescent channel detection of PN loading control. (D) Cross-reaction of the fluorescent PN signal with chemiluminescent signal of target antibody in non-transfected control sample.

The PN curve looks poor. Possibly, the dust in the capillary affected the performance of the PN fluorescent assay (Figure 7A) and Data analysis step 21d could not be performed.

### Potential solution

Repeat the Jess assay run with a new aliquot of a given sample.

### Problem 11

The oversaturation of signal in the fluorescence channel caused by the spiked peak of the PN curve (Figure 7B).

### Potential solution

In the case this spiked peak interferes with the size of the target protein, the sample run must be repeated. Else the PN Area can be manually reduced in the software (as in the Data analysis Optional step 21d) e.g., 20–180 or 30–180 etc., to exclude the oversaturated area from the analysis.

### Problem 12

The PN curve looks different from Figure 4E and the PN Corr. Area is less than 22,000 U (Figure 7C), imposing a risk that the detected signal is not in the linear range, which may reduce the reproducibility of the results (Data analysis step 21d).

### Potential solution

Repeat the Jess assay with a larger amount of the sample (refer to The Jess assay step 11b). If the total protein content of the stock sample is less than 1.5 mg/mL, reduce the volume of 1× Glo Lysis Buffer from 200 μL to 180 μL per sample (sample preparation step 4b).

### Problem 13

The cross-reaction of the PN kit reagent in the fluorescence channel and the antibody in the chemiluminescence channel Figure 7D leads to formation of non-specific peaks with larger sizes (Data analysis step 21d).

### Potential solution

If the cross-reaction peaks are larger than the target peak and well separated from it, they should not affect the analysis.

## Resource availability

### Lead contact

Further information and requests for resources and reagents should be directed to and will be fulfilled by the lead contact Leoš Shivaya Valášek (valasekl@biomed.cas.cz).

### Technical contact

Questions about the technical specifics of performing the protocol should be directed to and will be answered by the technical contact Leoš Shivaya Valášek (valasekl@biomed.cas.cz).

### Materials availability

Plasmid constructs generated in the associated primary research manuscript are available upon request.

### Data and code availability

• The data generated in this protocol and used for the preparation of Figures is available from the lead contact upon request.
• This protocol does not report an original code.
• Any additional information required to reanalyze the data reported in this protocol is available from the lead contact upon request.

## Acknowledgments

We are grateful to all past and present members of the Valasek lab for fruitful discussions. This work was supported by a Grant of Excellence in Basic Research (EXPRO 2019) provided by the Czech Science Foundation (19-25821X) and CZ.02.01.01/00/22_008/0004575 RNA for therapy by ERDF and MEYS (both to L.S.V.).

## Author contributions

A.M.S. and L.S.V. conceived and designed the project. A.M.S. carried out all experiments, analyzed the data, and wrote the first draft of the manuscript. A.H. and L.S.V. completed the manuscript preparation. L.S.V. supervised the project.

## Declaration of interests

The authors declare no competing interests.
